# Maternal zinc alleviates *tert*-butyl hydroperoxide-induced mitochondrial oxidative stress on embryonic development involving the activation of Nrf2/PGC-1α pathway

**DOI:** 10.1186/s40104-023-00852-1

**Published:** 2023-04-12

**Authors:** Liang Huang, Wei Gao, Xuri He, Tong Yuan, Huaqi Zhang, Xiufen Zhang, Wenxuan Zheng, Qilin Wu, Ju Liu, Wence Wang, Lin Yang, Yongwen Zhu

**Affiliations:** 1 State Key Laboratory of Livestock and Poultry Breeding, South China Agricultural University, Guangzhou, 510000 China; 2 Tongren Polytechnic College, Tongren, 554000 China; 3 Enping Long Industrial Co. Ltd, Enping, 529400 China

**Keywords:** Embryonic development, Maternal zinc, Mitochondrial function, Oxidative stress

## Abstract

**Background:**

Mitochondrial dysfunction induced by excessive mitochondrial reactive oxygen species (ROS) damages embryonic development and leads to growth arrest.

**Objective:**

The purpose of this study is to elucidate whether maternal zinc (Zn) exert protective effect on oxidative stress targeting mitochondrial function using an avian model.

**Result:**

In ovo injected *tert*-butyl hydroperoxide (BHP) increases (*P* < 0.05) hepatic mitochondrial ROS, malondialdehyde (MDA) and 8-hydroxy-2-deoxyguanosine (8-OHdG), and decreases (*P* < 0.05) mitochondrial membrane potential (MMP), mitochondrial DNA (mtDNA) copy number and adenosine triphosphate (ATP) content, contributing to mitochondrial dysfunction. In vivo and in vitro studies revealed that Zn addition enhances (*P* < 0.05) ATP synthesis and metallothionein 4 (MT4) content and expression as well as alleviates (*P* < 0.05) the BHP-induced mitochondrial ROS generation, oxidative damage and dysfunction, exerting a protective effect on mitochondrial function by enhancing antioxidant capacity and upregulating the mRNA and protein expressions of Nrf2 and PGC-1α.

**Conclusions:**

The present study provides a new way to protect offspring against oxidative damage by maternal Zn supplementation through the process of targeting mitochondria involving the activation of Nrf2/PGC-1α signaling.

**Graphical Abstract:**

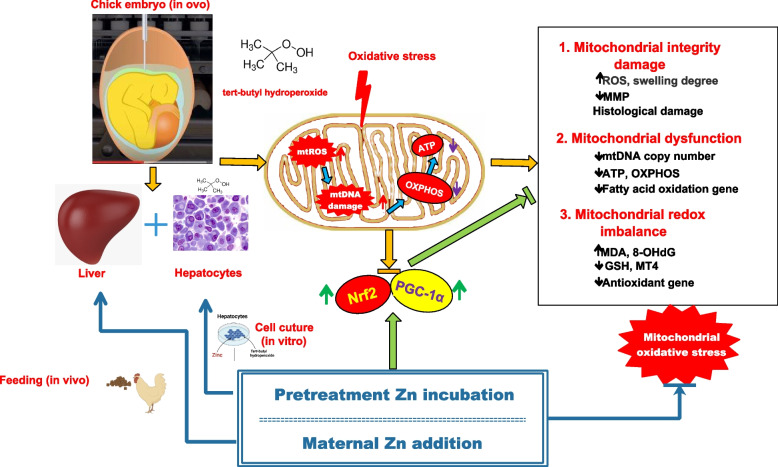

**Supplementary Information:**

The online version contains supplementary material available at 10.1186/s40104-023-00852-1.

## Introduction

Avian embryonic development is highly vulnerable to oxidative damage induced by both external and internal stressors, such as the cumulative effects of environmental contaminant exposure and incubation temperature changes, which are associated with abnormal development and embryotoxicity [1, 2]. Mitochondria as the major source of reactive oxygen species (ROS) are especially vulnerable to oxidative stress. Overproduction of mitochondrial ROS (mtROS) leads to oxidative damage of mitochondrial components and functions, such as the decrease of mitochondrial membrane potential (MMP) and mitochondrial DNA (mtDNA) copy number [3] and the defect of mitochondrial oxidative phosphorylation (OXPHOS) [4]. The energy in the form of adenosine triphosphate (ATP) generated from the mitochondrial electron transport chain is essential to maintain the embryonic development adequately. Therefore, mitochondrial dysfunction induced by oxidative stress leads to the cell death and embryonic growth retardation due to the insufficient energy supply [5].

*Tert*-butyl hydroperoxide (BHP) as a short-chain analog of lipid hydroperoxide has been used to induce oxidative damage on mitochondrial homeostasis and energy metabolism in the rat and cell [6, 7]. Thus, BHP toxic exposure by in ovo injection was selected to induce the mitochondrial oxidative stress of the chick embryo model, in order to mimic the effect of the external stressors that happened in practice during embryogenesis. Liver as a main and sensitive target organ for both energy metabolism and zinc (Zn) accumulation was also susceptible to oxidative stress. Many studies revealed that Zn as a cofactor of some distinct metalloenzymes is proposed to play a strong antioxidant role to restore mitochondrial oxidative damage [8]. Maternal Zn deficiency induces oxidative damage in liver resulting in embryonic development stagnation in the chick [9], duck [10] and mice [11]. Some studies have explored that maternal Zn supplementation could enhance the synthesis of hepatic metallothionein (MT) in offspring embryos [12], which is relevant in scavenging the excessive mitochondrial ROS generation under stressful conditions [13]. Therefore, it is speculated that maternal Zn supply serving as a mitochondrial antioxidant could exert a protective effect on offspring embryos subjected to the BHP-induced mitochondrial oxidative stress.

The capacity of Zn to regulate antioxidant protective responses is relevant in sustaining the redox homeostasis through various mechanisms [14]. On the one hand, the role of Zn in the upregulation of antioxidant protective responses could be achieved by enhancing the binding ability between redox-sensitive nuclear factor erythroid-2 related factor 2 (Nrf2) and antioxidant response elements [15, 16]. Apart from antioxidant agents, Zn has been proven to be essential for the transcription function of peroxisome proliferator-activated receptor γ coactivator-1α (PGC-1α) pathway in mitochondrial biogenesis, oxidative phosphorylation and energy metabolism of primary human endometrial stromal cells [17]. Nevertheless, there is no evidence that Zn supplementation exhibits a potential protective effect on oxidative stress targeting mitochondrial function via PGC-1α signaling. The avian embryos are much easier to maintain and manipulate than most other vertebrate species, making the avian embryos an ideal model organism to investigate embryonic development under different maternal nutritional levels and stressed conditions. The purpose of this study is to detect the mitochondrial oxidative stress induced by in ovo BHP injection and to assess the ameliorative effect of maternal Zn supplementation on mitochondrial oxidative stress via Nrf2/PGC-1α pathway in liver using avian embryo as a test model.

## Methods and materials

The animal care and use protocol was approved by the Animal Care and Use Committee of South China Agricultural University (SCAU-10564), and the study was conducted following the Regulations for the Administration of Affairs Concerning Experimental Animals.

### Preparation of BHP solutions and in ovo injection procedure

The *tert*-butyl hydroperoxide (458139, Sigma, Westborough, MA, USA) was dissolved in PBS solution to prepare a stock solution containing 1200 μmol/L BHP, which was subsequently diluted with PBS to make injection solutions containing 600, 300, and 150 μmol/L BHP, respectively. All solutions were filtered using a 0.22-μm acetate filter (16541-K, MSI®, Westborough, MA, USA). The injection was carried out at embryonic day 14 (E14), and the procedure of in ovo injection according to the description by Sun et al. [18]. Under the candle, the outline of the air cell was drawn using a pencil. The embryo, which appeared as a dark floating silhouette and the head like a dark spot, was located. Before injections, the eggs were removed from the incubator and candled for viability. The fertilized eggs were disinfected with 75% ethanol in the needle insertion region before injection. The sterile disposable 25.0 mm × 0.6 mm needle was attached to a 1-mL syringe, which was replaced after each egg injection. The holes were sealed with medical adhesive tape (1.0 cm × 1.0 cm) immediately after injection, and the eggs from each replicate of each treatment were placed on the same egg tray, which were maintained at an incubation temperature of 38.0 ± 0.5 ℃ and a relative humidity of 55% ± 3% until embryonic day 18 (E18). Then, all eggs were transferred to hatching crates and moved to hatchers. The hatcher was set at a temperature of 36.5 ± 0.5 ℃ with a related humidity of 75% until embryonic day 21 (E21).

### Inducing mitochondrial oxidative stress in embryos by in ovo BHP injection (in ovo, Exp. 1)

For the purpose of inducing a chick embryonic model of mitochondrial oxidative stress, the effect of graded concentrations of in ovo injected BHP were evaluated on the embryonic development, mitochondrial structure and function in Exp. 1. A total of 720 fertilized eggs (breed: Qingyuan partridge chicken) with similar weights were randomly divided into 6 treatments with 6 replicates per treatment and 20 eggs per replicate. According to the recommended the injected dosages of BHP in the rat [19], all hatched eggs were injected with 0.1 mL PBS (G4202, Servicebio, Wuhan, Hubei, China) as control or 0.1 mL solutions of 150, 300, 600, or 1200 μmol/L BHP per kg egg weight through yolk sac at E14, respectively. After in ovo injection, eggs from each replicate of each treatment were placed on the same egg tray, and incubated at 38.0 ℃ with a relative humidity of 55%. At embryonic day 15 (E15), the eggs were candled for viability, and any dead embryos were counted. At embryonic day 22 (E22), embryonic mortality was calculated as a percentage of dead embryos in the total number of injected eggs for each replicate of per treatment. From E15 to E20, 12 embryos from each treatment were sampled for further analysis.

### Animal feeding experiments and diets (in vivo, Exp. 2)

To investigate whether maternal Zn nutrition could alleviate the deleterious effect of mitochondrial oxidative stress on embryonic development, a completely randomized design including that the embryos from the two maternal groups were subjected to in ovo injection treatments of PBS and 600 μmol/L BHP, respectively in Exp. 2. The two maternal dietary Zn treatments were a semi-purified basal diet without Zn supplementation (Zn-deficient control diet, Con group) and was supplemented with 220 mg of Zn/kg diet as Zn sulfate (Zn-adequate diet, Zn group). The supplemental level of 220 mg Zn/kg of diet as Zn sulfate was recommended by the local Enterprise Agricultural Industry Standard [20] to meet Zn requirements of yellow-feathered broiler breeders during the laying period.

The feeding experimental procedures were described in detail in our previous study [9]. In brief, a total of 144 21-week-old yellow-feathered broiler breeders (breed: Qingyuan partridge chicken) were obtained from a commercial farm (Jilong Group, Enping, Guangdong, China) and housed in the caged system for an adaptation period of 21 to 28 weeks old. Subsequently, all female breeders followed by a semi-purified diet (11.99 MJ ME/kg, 158 g CP/kg, 8.8 g lysine/kg, 7.3 g methionine + cysteine/kg, 34.0 g calcium/kg, 4.6 g available phosphorus/kg) containing 26.34 mg Zn/kg to deplete Zn stores from 29 to 30 weeks of age. After that, laying broiler breeders were randomly allotted into 2 dietary Zn levels with 6 replicates of 12 birds per replicate for the experimental period of 31 to 36 weeks old. The composition and nutritional levels of the basal diet were shown in Table S1. The analyzed values of Zn contents in Con and Zn diets were 26.34 and 245.67 mg/kg, respectively. At the end of experimental period, feed consumption and egg weight were measured weekly. Twelve eggs from each treatment (2 per replicate) were collected on the last day of experimental period for egg yolk Zn analysis. Then, a total of 120 fertilized eggs from each treatment were divided into two in ovo treatments of the PBS and BHP injection on E14, respectively. The incubation and in ovo injection procedures and sample collection were performed as described in Exp. 1.

### Hepatocellular carcinoma cell line culture and treatments (in vitro, Exp. 3)

Chicken hepatocellular carcinoma cell line (LMH) were obtained from ATCC (Manassas, Virginia, USA) and cultured as described previously [21]. Briefly, cells were seeded at 3 × 10^5^ cells/well in 96-well culture plates (3599, Corning, New York, USA), which were allowed to adhere for 24 h. The cells were incubated at 37 ℃ in a humidified atmosphere of 5% CO_2_ at 95% humidity, were maintained with their media changed three times weekly. After 11 d, cells were rinsed by PBS and incubated with Zn-free medium for 24 h. In order to obtain the noncytotoxic concentrations of BHP-induced mitochondrial oxidative stress firstly, cells were treated with H_2_O_2_ as control and with BHP at various concentrations (0, 10, 20, 50, 100, 200, and 500 μmol/L) [22]. The cells were treated for 48 h and harvested for subsequent analysis. Then, in order to verify the protective effect of Zn on BHP-induced oxidative stress in cells (Exp. 3), the cells were pretreated in the presence or absence of Zn for 12 h, and treated with BHP for 2 h afterwards. The Zn concentration of 50 μmol/L was selected according to the published study [18]. The cells were treated for 14 h and harvested for subsequent analysis. The CCK8 solution (10 μL) was added into each well, and the cells were incubated at 37 ℃ for 2 h. The color intensity was determined using a microplate reader (model 550, BioRad, Richmond, Virginia, USA) at 450 nm. Cell viability was expressed as the percentage relative to the normalized average of the control.

### Isolation of hepatic mitochondria

The liver samples were cut into small pieces and placed in 10 vol cold Buffer A (1 mg/mL fatty acid-free BSA, 75 mmol/L sucrose, 225 mmol/L mannitol, 10 mmol/L N-2-hydroxyethylpiperazine-N-ethane-sulphonicacid (HEPES), and 0.1 mmol/L ethylene glycol tetraacetic acid (EGTA), pH 7.4), which was supplemented with phosphatase and protease inhibitors (ab201119, Abcam, Boston, Massachusetts, USA). After that, sample tissue was homogenized with a Teflon Dounce homogenizer, followed by differential centrifugation to isolate mitochondria (700 × *g* for 15 min and then 10,000 × *g* for 15 min at 4 ℃). The resultant supernatant containing cytoplasm was collected for the analysis related to oxidative damage. The sediment containing mitochondria were subsequently resuspended within Buffer B (10 mmol/L HEPES, 0.1 mmol/L EGTA, and 395 mmol/L sucrose, pH 7.4). Total proteins of cytoplasm and mitochondria solutions were measured by the BCA method using the total protein quantitative assay kit (A045-4–2, Nanjing Jiancheng Institute of Bioengineering, Jiangsu, China).

### Determination of Zn concentration, ROS, MMP, swelling degree and ATP content

The diets and egg yolk samples were collected to analyze Zn contents, which were measured using an inductively coupled plasma emission spectroscope (IRIS Intrepid II, Thermal Jarrell Ash, Waltham, Massachusetts, USA) after wet digestions with HNO_3_ and HClO_4_ as described previously [9]. Intracellular ROS of liver samples and cells was measured by using a nonfluorescent probe, namely, 2,7-dichlorodi-hydrofluorescein diacetate (DCFH-DA; CT0045, Leagene Biotechnology, Beijing, China), which can freely penetrate the intracellular matrix and be oxidized by ROS to fluorescein dichloride (DCF). The fluorescence intensity of DCF reflecting cellular level of ROS was measured by excitation and emission filters settings at 485 and 525 nm, respectively. The MMP was measured with MMP assay kit with JC-1 (5 μg/mL, DF0080, Leagene Biotechnology, Beijing, China) as described in the manufacturer’s instructions. In the presence of a high MMP, JC-1 forms J-aggregates which emit red fluorescence, while JC-1 monomeric form emits green fluorescence at low MMP. Both colors were detected using a fluorescence microplate reader (FLx800^TM^, BioTek® Instruments, Inc., Winooski, VT, USA). The mitochondrial swelling degree (MSD) was measured with the mixed solution of 0.5 μmol/L vitamin C and 5 μmol/L FeSO_4_ at 24 ℃ for 20 min. The absorbance was measured at a wavelength of 520 nm using a Multiskan GO microplate (Thermo Fisher Scientific, Vantaa, Finland). According to the manufacturer's protocol (A095-1–1, Nanjing Jiancheng Institute of Bioengineering, Jiangsu, China), the ATP level was analyzed by luminescence detection with FluoStar using a fluorescence microplate reader.

### Determination of related indices of antioxidant status

The malondialdehyde (MDA) was determined by thiobarbituric acid colorimetric method (A003, Nanjing Jiancheng Institute of Bioengineering, Jiangsu, China). The contents of 8-hydroxy-2-deoxyguanosine (8-OHdG), MT4 and GSH were measured using commercial kits according to the manufacturer’s instructions (H165, A006-2–1 and H132, Nanjing Jiancheng Institute of Bioengineering). The concentrations of total protein in cytoplasm and mitochondria were determined as described before and all indices were expressed as units per milligram protein.

### Targeted metabolomics analysis of energy metabolism

Targeted metabolomics analysis was performed by Aptbiotech Co., Ltd. (Shanghai, China) using GC–MS method. The samples were thawed at 4 ℃, and 100 μL aliquots were mixed with 400 μL cold methanol/acetonitrile (1:1, v:v) to precipitate protein. The mixture was centrifuged for 20 min (14,000 × *g*, 4 ℃). And then the supernatant was dried in a vacuum centrifuge. For liquid chromatography-mass spectrometry (LC–MS) analysis, the samples were redissolved in 100 μL acetonitrile/water (1:1, v:v). After vortex for 1 min, the tubes were centrifuged for 15 min (14,000 × *g*, 4 ℃), and the supernatants were collected for LC–MS analysis. The analysis was carried out using an ultra-high-performance liquid chromatography system (1290 Infinity LC, Agilent Technologies, Palo Alto, CA, USA) coupled with to a QTRAP (5500, AB Sciex, Framingham, MA, USA).

### Histological analysis

The histological study was performed as described previously [19]. Briefly, embryonic liver samples were fixed in 10% formalin, dehydrated, embedded in easin, sectioned at 5 μm and stained with hematoxylin and eosin (HE staining). For the ultrastructural study, small tissue fragments were fixed in 2.5% glutaraldehyde in 0.1 mol/L phosphate buffer at 48 ℃ for 1 h, washed in 0.1 mol/L phosphate buffer, and embedded in agar chips, which were further postfixed in 1% osmium tetroxide in 0.1 mol/L phosphate buffer, pH 7.2. they were dehydrated and then embedded in Araldite/Epon (Electron Microscopy Sciences, Washington, USA) and sectioned into semithin slices, that were stained with toluidine blue. The ultrathin sections were contrasted with uranyl acetate and lead citrate and then observed via transmission electron microscopy at 200 kV.

### Quantitative real time-qPCR (RT-qPCR) analysis

Gene expression analysis was performed using RT-qPCR as described previously [9]. Total RNA from samples was isolated with EZgeneTM Tissue RNA kit (BN20537, Baierdi, Beijing, China) and transcribed into cDNA by using a reverse transcription kit using SYBR Green (RR042, TaKaRa, Osaka, Japan) and then RT-qPCR of gene mRNA expression was conducted using SYBR Green Quantitative PCR kit (RR037, TaKaRa, Osaka, Japan). The expression of each gene was normalized to that of the reference gene of β-actin, and the results were presented relative to the control group according to the 2^−ΔΔCt^ method. The primers used were listed in Table S2.

### Mitochondrial DNA copy number quantification

Mitochondrial DNA copy number was determined as a marker of mitochondrial abundance using RT-qPCR method as described previously [23]. Total DNA was isolated by TIANamp Genomic DNA Kit (D1700, Solarbio, Beijing, China) according to the manufacturer’s instruction. The concentration of DNA in the extracts was measured by a NanoDrop 2000 Spectrophotometer (Thermo Fisher Scientific, Waltham, MA, USA). DNA primer was designed to detect ATP6 as the makers for mtDNA (Table S2). The quantification of mtDNA copy number was normalized to that of the reference gene of glucagon gene.

### Western blot

Tissue and cell samples were washed with PBS once and lysed with Western blot lysis buffer (0.25 mol/L Mannitol, 0.05 mol/L Tris, 1 mol/L EDTA, 1 mol/L EGTA, 1 mmol/L DTT, 1% Triton-X) containing Complete Mini Protease Inhibitor Cocktail (ab201119, Abcam, Boston, MA, USA). The extracts were centrifuged at 10,000 × *g* at 4 ℃ for 15 min to remove insoluble cell debris. The total amount of protein was determined using the Pierce BCA Protein Assay Kit (Perbio Science, Bonn, North Rhine-Westphalia, Germany). On basis of Western blot analysis, 60 µg of protein were loaded on a 1.5-mm SDS-Gel and blotted onto a PVDF membrane at 260 mA for 1.5 h. Incubation with primary antibody at 4 ℃ was performed overnight. The following primary antibodies were used according to Table S3. After incubation with a proper secondary HRP-labelled antibody (Vector Laboratories, Burlingame, CA, USA), western blot signals were detected via chemiluminescence with ChemiDoc Imaging Systems (Bio-Rad, Hercules, CA, USA).

### Statistical analysis

Data were presented as the mean ± SEM. GraphPad Prism was applied for graphing and statistical analysis (version 5, GraphPad Software). The results from two groups of experiments were analyzed by Student's *t*-test, while those of more than two groups were analyzed by a one-way ANOVA followed by Tukey's post hoc test to compare all the groups.

## Results

### In ovo BHP injection induced hepatic mitochondrial oxidative damage (Exp. 1)

**Fig. 1:**
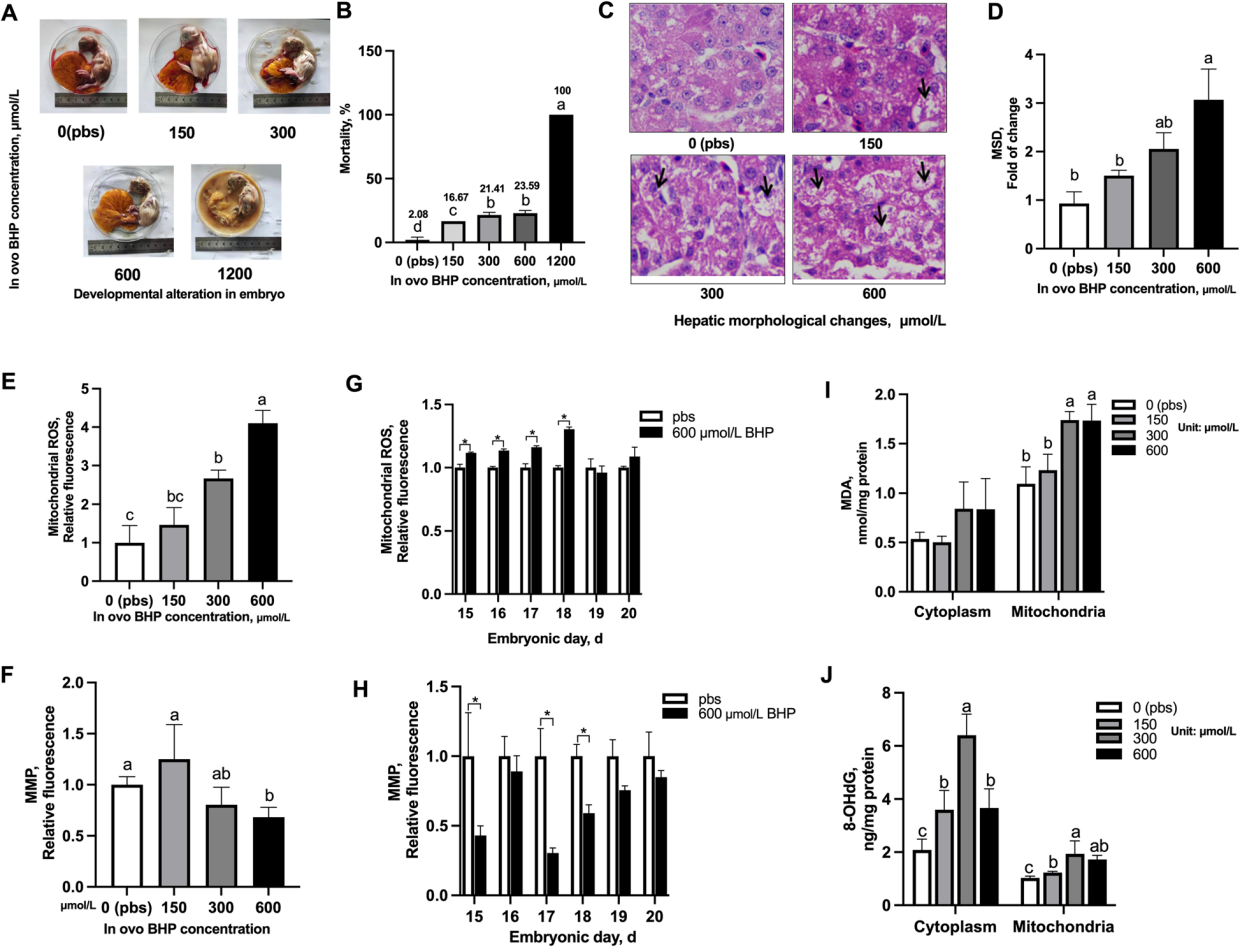
In ovo BHP injection increases mortality, mitochondrial ROS accumulation and oxidative damage in embryonic liver. **A** Developmental alteration in chick embryo in response to in ovo injected BHP levels after 24 h. The BHP concentrations of in ovo injection were 0 (pbs), 150, 300, 600, and 1200 μmol/L BHP per kg egg weight. **B** Embryonic mortality induced by BHP in ovo injection in a dose dependent manner. The mortality was up to 100% observed at 1200 μmol/L BHP group. **C** Representative hepatic morphological changes in embryo by HE staining. Black arrows represent the swollen and damaged hepatic tubule and glomerulus (black arrows). All micrographs 200 × magnification, hematoxylin/eosin staining. **D** Increase of hepatic MSD induced by BHP in ovo injection. **E** Hepatic mtROS accumulation induced by in ovo BHP injection. **F** Decrease of hepatic MMP by in ovo BHP injection. **G**–**H** Aged changes of hepatic mitochondrial ROS and MMP induced by BHP in ovo injection. **I** Lipid peroxidation assessed by MDA production in isolated cytoplasm and mitochondria. **J** The mtDNA damage assessed by 8-OHdG production in isolated cytoplasm and mitochondria. Graph bars in **B**, **D**, **E**, **G**, **H**, **I** and **J** marked with different letters on top represent statistically significant results (*P* < 0.05) based on Tukey's post hoc one-way ANOVA analysis, whereas bars labelled with the same letter correspond to results that show no statistically significant differences. The results from **G** and **H** were analyzed using unpaired two-tailed Student's *t*-test (^∗^*P* < 0.05). Data are mean ± SEM (*n* = 3 or 4)

The dose effect of in ovo BHP injection on embryonic development was observed, and the injected 150, 300, 600 and 1200 μmol/L BHP groups (Fig. 1A) presented pronounced morphological deformities, such as reduced size, twisted neck, folding of legs, and marked inhibition of the vasculature compared with normal embryos in 0 μmol/L BHP group (Fig. 1A). After 24 h, with the increase of BHP-injected doses, the embryonic mortality (Fig. 1B), hepatic MSD (Fig. 1D) and mitochondrial ROS (Fig. 1E) in response to increased linearly (*P* < 0.05). The mortality of embryos of 1200 μmol/L BHP group was up to 100%. HE staining showed notable histological damage at the junction of medulla and cortex in BHP injection groups, including severe vacuolation and increased focal tubular necrosis. There was a decrease in MMP and an increase (*P* < 0.05) in mitochondrial MDA level in 600 μmol/L BHP group compared with 0 and 150 μmol/L BHP groups. There was an increase (*P* < 0.05) in 8-OHdG levels of cytoplasm and mitochondria in 300 and 600 μmol/L BHP groups compared with 0 μmol/L BHP group. In addition, the increase of hepatic mitochondrial ROS and MMP (*P* < 0.05) caused by the injection of 600 μmol/L BHP were observed until E18. Thus, in ovo BHP injection impaired the integrity of hepatic mitochondria and 600 μmol/L BHP group was selected to induce oxidative damage for further experiments.

### In ovo injected BHP induced hepatic mitochondrial dysfunction and redox imbalance (Exp. 1)

**Fig. 2:**
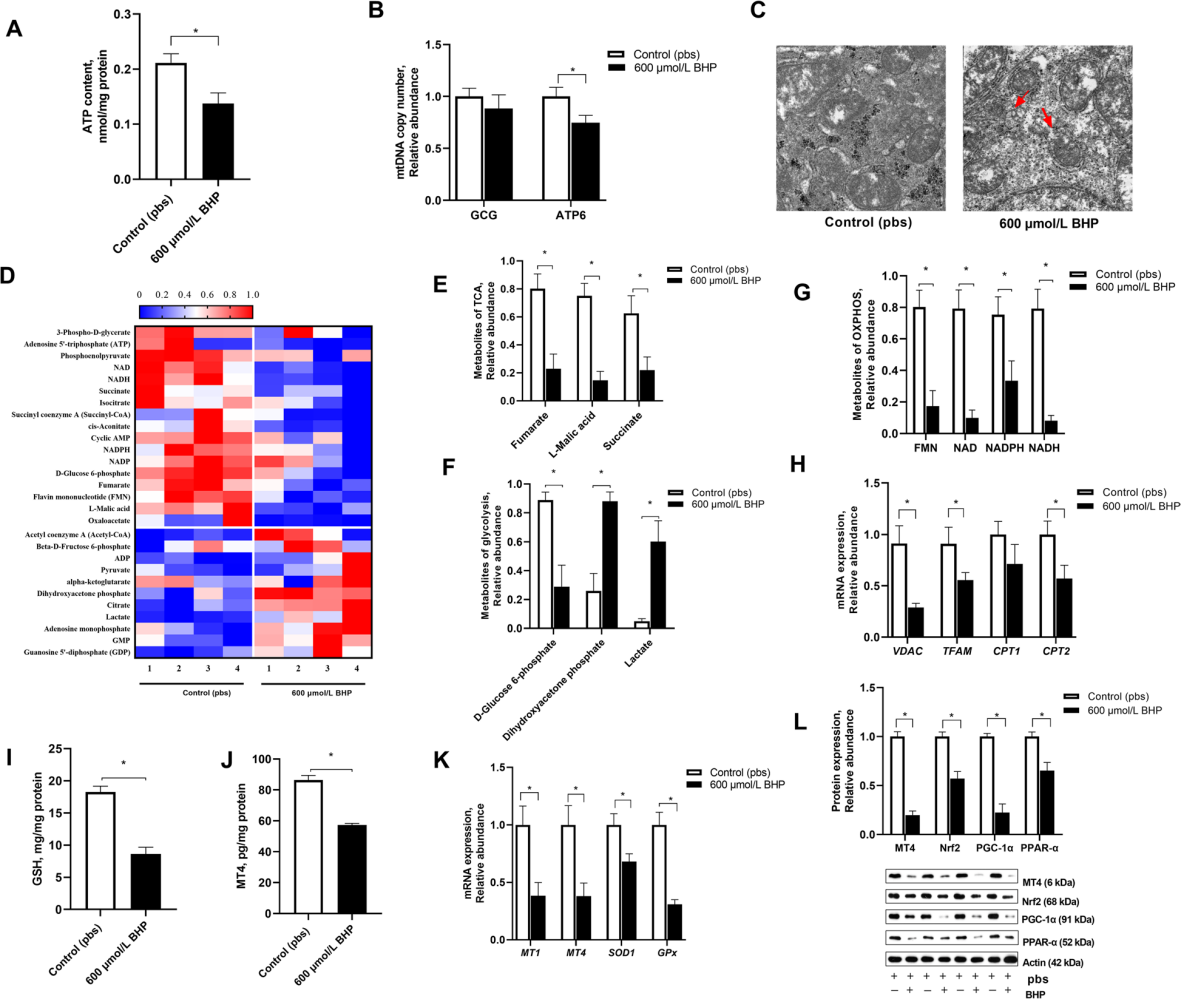
In ovo injected BHP induced hepatic mitochondrial dysfunction and redox imbalance in embryo. The control and BHP groups were treated with either pbs or 600 μmol/L BHP by in ovo injection. **A** Hepatic ATP content. **B** Hepatic mtDNA copy number. **C** Representative electron microscopy micrographs of mitochondrial ultrastructure. The mitochondria from the BHP treated embryo was smaller with a condensed mitochondrial membrane density (red arrows). **D**–**G** The abundance heatmap of metabolites for energy metabolism by targeted metabolomic analysis (**D**) The relative abundance of metabolites related to TCA (**E**) and oxidative phosphorylation (**E**) and glycolysis (**F**). **H** Hepatic gene mRNA expression of *CPT1, CPT2, VDAC,* and *TFAM* related to mitochondrial function. **I**–**J** Hepatic GSH and MT4 contents in mitochondria. **K** Hepatic mRNA expression of *MT1, MT4, Gpx,* and *SOD1* related to the antioxidant ability. **L–M** Representative band of hepatic MT4, Nrf-2, PGC-1α, and PPAR-α protein expression by Western blot assay. The data were analyzed using unpaired two-tailed Student's *t*-test (^∗^*P* < 0.05). Data are mean ± SEM (*n* = 4–6)

Given the close relationship between mitochondrial structure and function, this paper investigated whether there was mitochondrial dysfunction mediated BHP-induced hepatic oxidative stress in embryo. Compared with the control group, 600 μmol/L BHP injection group reduced (*P* < 0.05) the mitochondrial ATP content (Fig. 2A) and the mtDNA copy number of ATP6 (Fig. 2B) in embryo. Histological staining with TEM indicated that the liver of BHP injection group exhibited smaller (*P* < 0.05) mitochondria and condensed mitochondrial membrane densities than that of the control group (Fig. 2C). Furthermore, this paper compared the relative abundance of targeted metabolomic analysis for energy metabolism between the control and 600 μmol/L BHP groups (Fig. 2D). Compared with the control group, BHP injection decreased (*P* < 0.05) the abundances fumarate, *L*-malic acid and succinate related to TCA (Fig. 2E) and FMN, NAD, NADH, and NADPH related to OXPHOS (Fig. 2G) as well as increased (*P* < 0.05) the abundances of *D*-glucose, 6-phosphate, dihydroxyacetone phosphate, and lactate related to glycolysis (Fig. 2F). Compared with the control group, the contents of mitochondrial GSH and MT4 was decreased (*P* < 0.05) following 600 μmol/L BHP injection (Fig. 2I-J), so did the mRNA expression of genes in relate to fatty acid oxidation (*VDAC, TFAM, CPT-1* and *CPT-2*; Fig. 2H) and antioxidant capacity (*MT1, MT4, SOD1* and *Gpx*; Fig. 2K). Western blot analysis showed that BHP injection downregulated (*P* < 0.05) the protein expression of MT4, Nrf2, PGC-1α and peroxisome proliferators-activated receptor-α (PPAR-α) (Fig. 2L). Thus, in ovo injected BHP disturbed hepatic mitochondrial function and triggered redox imbalance.

### Maternal Zn addition attenuated in ovo injected BHP-induced mitochondrial dysfunction in embryo (Exp. 2)

**Fig. 3:**
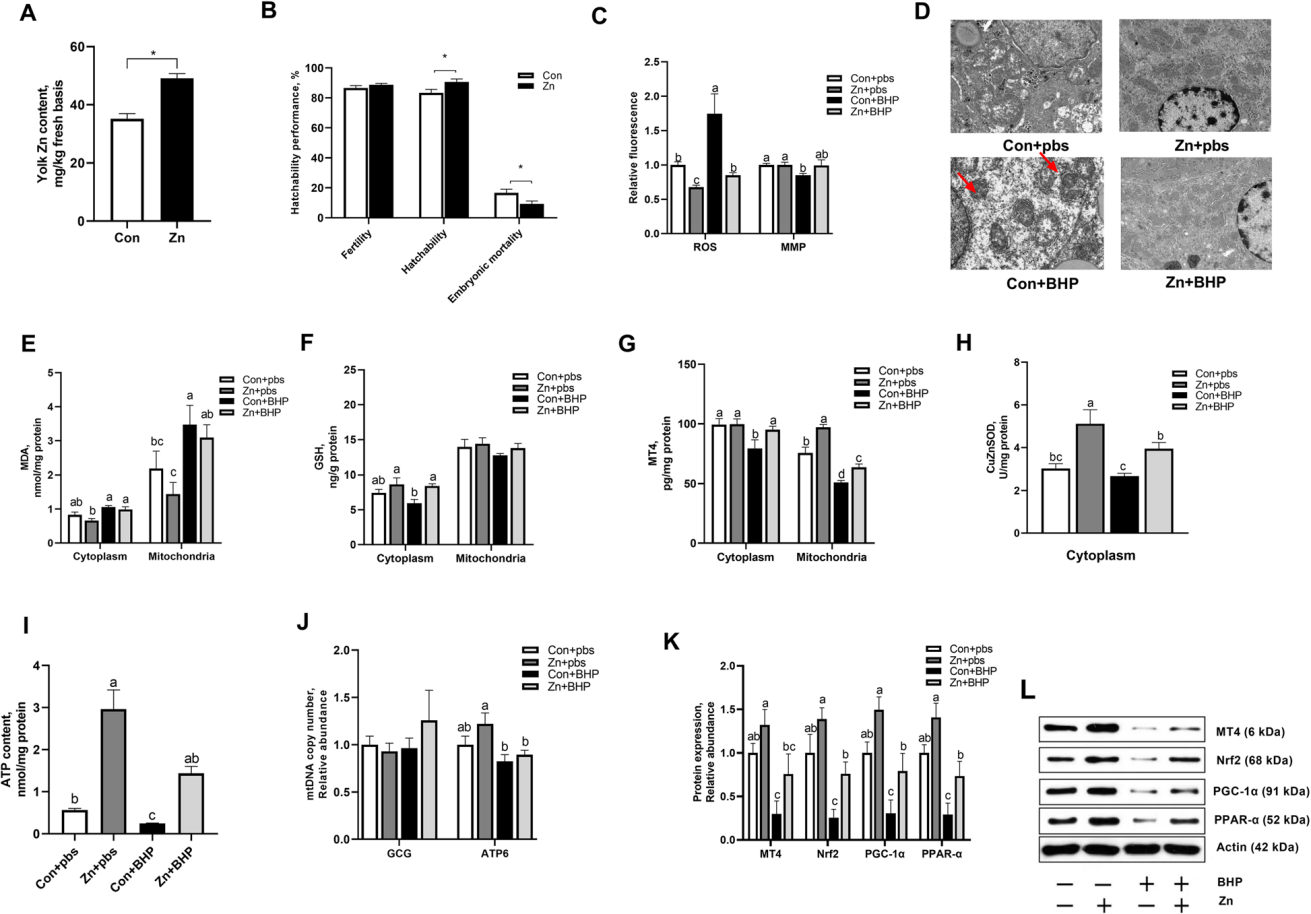
Maternal Zn addition attenuated in ovo injected BHP-induced mitochondrial dysfunction in embryo. The maternal Con and Zn groups diets were supplemented with either 0 or 220 mg Zn/kg diet for female broiler breeders. The embryos from Con and Zn groups were subjected to in ovo injection of either pbs or 600 μmol/L BHP on E14. **A** Effect maternal Zn addition on egg yolk Zn content. **B** Effect maternal Zn addition on hatchability performance. **C** Effect maternal Zn addition and in ovo injected BHP treatment on mitochondrial ROS and MMP. **D** Representative electron microscopy micrographs of mitochondrial ultrastructure. **E**–**G** Effect maternal Zn addition and in ovo injected BHP treatment on MDA, GSH, and MT4 contents in isolated cytoplasm and mitochondria. **H** Effect maternal Zn addition and in ovo injected BHP treatment on CuZnSOD activity in isolated cytoplasm. **I**–**J** Effect maternal Zn addition and in ovo injected BHP treatment on hepatic ATP content and mtDNA copy number. **K** and **L** Effect maternal Zn addition and in ovo injected BHP treatment on hepatic MT4, Nrf-2, PGC-1α, PPAR-α protein expressions. Graph bars in A and B were analyzed using unpaired two-tailed Student's t-test (^∗^*P* < 0.05, *n* = 6), while graph bars in **C**, **E**, **G**, **H**, **I** and **J** marked with different letters on top represent statistically significant results (*P* < 0.05, *n* = 4–6) based on Tukey's post hoc analysis, whereas bars labelled with the same letter correspond to results that show no statistically significant differences. Data were mean ± SEM

In the feeding experiment, compared with the Con group, Zn group increased (*P* < 0.05) Zn content in egg yolk (Fig. 3A) and reduced (*P* < 0.05) embryonic mortality (Fig. 3B), but did not influence the laying performance of broiler breeders (Table S4). This paper further determined whether maternal Zn nutrition could reduce (*P* < 0.05) mitochondrial oxidative stress induced by in ovo BHP injection in embryo. The Zn group resulted in the decrease of hepatic mitochondrial ROS (*P* < 0.05) compared with the Con group (Fig. 3C). Notably, maternal dietary Zn addition reversed the increase of hepatic mitochondrial ROS and MMP (*P* < 0.05) and structural damage of embryos induced by BHP injection (Fig. 3D). There was a decrease (*P* < 0.05)) in MDA content (Fig. 3E) and an increase (*P* < 0.05) in MT4 content (Fig. 3G) in hepatic mitochondria and cytoplasm as well as GSH content (Fig. 3F) and CuZnSOD activity (Fig. 3H) in cytoplasm in Zn group compared with Con group. Moreover, Zn group efficiently increased (*P* < 0.05) the hepatic ATP and MT4 contents and CuZnSOD activity of the embryos subjected to in ovo BHP injection to normal levels. Notably, maternal Zn supplementation ameliorated the decreased (*P* < 0.05) MT4, Nrf-2, PGC-1α, and PPAR-α proteins expression of embryos subjected to BHP-induced oxidative stress.

### The protective effect of Zn on BHP-induced oxidative stress in LMH (Exp. 3)

**Fig. 4:**
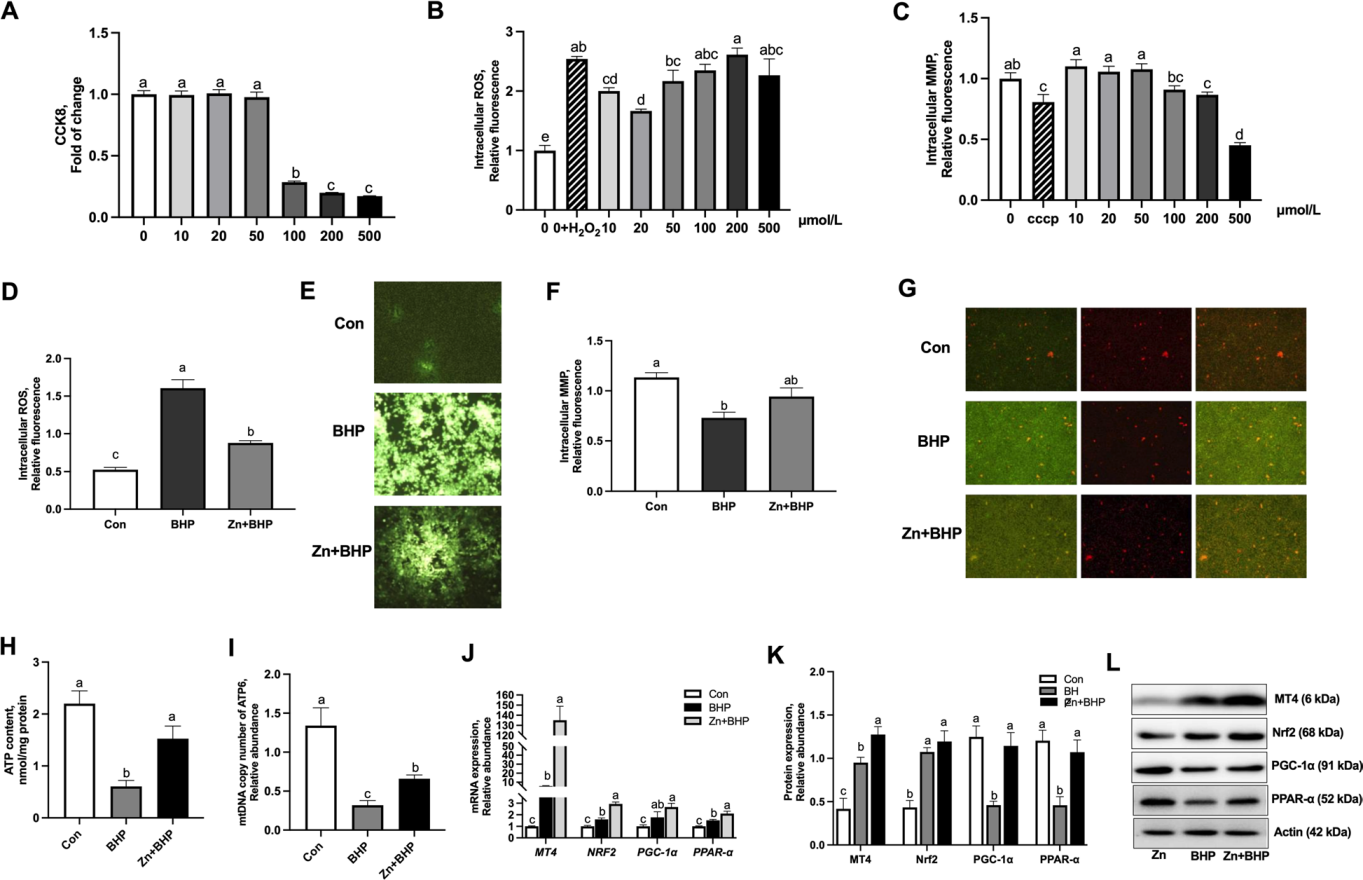
The protective effect of Zn against BHP-induced oxidative stress in hepatocytes. **A** The cell viability assessed by CCK8 in response to the BHP incubation at various concentrations. **B**–**C** The mitochondrial ROS (**B**) and MMP (**C**) in response to the incubation BHP at various concentrations. **D** Effect of Zn pretreatment on the level of intracellular ROS generation by 100 μmol/L BHP incubation. **E** Intracellular ROS generation was measured by DCFH-DA staining. **F** Effect of Zn pretreatment on intracellular MMP in hepatocytes subjected to 100 μmol/L BHP incubation. **G** Intracellular ROS was measured by JC-1 staining. In the presence of a high MMP, JC-1 forms J-aggregates that emit red fluorescence, while JC-1 monomeric form emits green fluorescence at low MMP. **H**–**I** Effect of Zn pretreatment on intracellular ATP content and mtDNA copy number of ATP6 in hepatocytes subjected to 100 μmol/L BHP incubation. **K**–**L** Effect of Zn pretreatment on MT4, Nrf-2, PGC-1α, PPAR-α protein expression in hepatocytes subjected to 100 μmol/L BHP incubation. Graph bars in **A** and **B** were analyzed using unpaired two-tailed Student's *t*-test (^∗^*P* < 0.05), while graph bars in **C**–**L** marked with different letters on top represent statistically significant results (*P* < 0.05) based on Tukey's post hoc one-way ANOVA analysis, whereas bars labelled with the same letter correspond to results that show no statistically significant differences. Data are mean ± SEM, *n* = 4–6

To explore the convincing evidence of the protective role of Zn nutrition on oxidative stress, the effect of various incubation concentrations of BHP from 0 to 500 μmol/L on oxidative damage of hepatocytes. The BHP treatments of 100, 200 and 500 μmol/L concentrations increased (*P* < 0.05) intracellular ROS concentration (Fig. 4B) and decreased cell viability (Fig. 4A), and MMP (Fig. 4C). The fluorescent assay revealed that Zn pretreatment mitigated the adverse effects (*P* < 0.05) on intracellular ROS (Fig. 4D–E) and MMP (Fig. 4F–G) in hepatocytes incubated with 100 μmol/L BHP. The intracellular ATP content (Fig. 4H) and mtDNA copy number (Fig. 4I) reduced (*P* < 0.05) by 100 μmol/L BHP incubation, but increased by Zn pretreatment. Notably, pretreatment of Zn incubation increased (*P* < 0.05) mRNA and protein expressions of Nrf2, MT4, PGC-1α, and PPAR-α in hepatocytes subjected to 100 μmol/L BHP incubation (Fig. 4J–K), which further confirmed that Zn supplementation ameliorated the BHP-induced oxidative damage by upregulation of MT4 expression and Nrf2/PGC-1α pathway in vitro.

## Discussion

Embryos are susceptible to oxidative stress, which exposure to excessive ROS has been implicated in the arrested development of mammalian embryos in vitro [1, 24]. Mitochondria, as the reaction pathway of the electron transport chain that generates ATP, are considered to be the main source of ROS during embryonic development [5], and the excessive production of ROS can damage cellular macromolecules, including nucleic acids, phospholipids, and proteins and then induce cell death [25]. As reported previously [19], BHP-induced oxidative damage has an important impact on mitochondrial homeostasis, which is selected to induce the mitochondrial oxidative stress of the chick embryo model with the in ovo injection on E14 in our study. Around the day of E14, mitochondrial aerobic respiration is becoming the major energy production for embryonic development since oxygen was available from the chorioallantoic membrane fully developed [26]. Due to the atmospheric oxygen of more saturated fatty acids, the accumulation of polyunsaturated fatty acids in the tissue lipids increased the susceptibility of the chick embryo to oxidative stress in the particularly critical period [27]. A significant depletion in MMP was noted at 600 μmol/L BHP dose associated with the overproduction of mitochondrial ROS and MSD. In addition, the injection of 600 μmol/L BHP has a continuous effect on the production of mitochondrial ROS and the decrease of MMP on embryonic development until E18. The increasing injected doses of BHP pronounces the lipid peroxidation and the mitochondrial DNA damage, as evidenced by the increased mitochondrial MDA and 8-OHdG accumulation, which are in agreement with the previous report in the rat [28]. Mitochondrial lipid peroxidation products can contribute to impairing the structural integrity of mitochondria by interacting with lipid moieties in the membrane. In order to confirm the underlying mode of action induced by BHP, this paper finds that a similar dose–response of the increased intracellular ROS level and reduced MMP in manner were confirmed in hepatocytes, which is consistent with the previous reports in hepatocytes in vitro [29, 30]. The experimental results present that the oxidative damage induced by the increase of BHP level is related to a dose-dependent linear increase in embryo mortality and embryo toxicity. The mortality reaches 100% by in ovo 1200 μmol/L BHP after 24 h, respectively. Similar results were confirmed in vivo and in vitro studies [31, 32] that embryos under higher oxygen concentration induce the increase of the intracellular ROS in mitochondria, which can greatly affect developmental fate. It is noteworthy that the reductions of hepatic GSH and MT4 contents and the gene expression of *MT1, MT4, SOD1* and *Gpx* by BHP injection one of the causes for the induction of mitochondrial oxidative damage and dysregulation. To sum up, this paper speculates that the imbalance between the production of ROS and the scavenging of the antioxidant system could be an important reason for the arrest of the development of chick embryos.

On the other hand, oxidative stress is associated with mitochondrial dysfunction by altering intracellular energy metabolism [33]. This work demonstrates that BHP induces the mitochondrial fragmentation with cristae effacement and autophagosomes appeared in the mitochondrial ultrastructure, which would be triggered by the loss of MMP, mitochondrial decoupling and oxidative damage. The mitochondrial dynamics alterations induced by 600 μmol/L BHP reduces the ATP production due to the mitochondrial permeability transition induction, which is confirmed in rat hepatocytes in vitro [34]. Moreover, targeted metabolomics analysis reveals that 600 μmol/L BHP in ovo inhibited the TCA cycle characterized by the decreased fumarate, *L*-malic acid and succinate, but enhanced the glycolysis characterized by the increased *D*-glucose, 6-phosphate, dihydroxyacetone phosphate, and lactate. The bio-maker of mtDNA copy is more vulnerable due to the absence of the protection of the electron transport chain [35]. The results indicate that oxidative damage reduces the mtDNA copy number of ATP6 associated with the impaired mitochondrial biosynthesis. Since mitochondrial oxidative stress leads to mitochondrial fragmentation via the imbalance of mitochondrial fission–fusion [36]. Oxidative mtDNA lesion contributes to defects in OXPHOS, and thus provides insufficient energy requirements for embryo development. Consistently, 600 μmol/L BHP injection downregulated the mRNA abundance of *VDAC, TFAM, CPT-1* and *CPT-2* related to fatty acid oxidation, which confirms the inhibition of cellular respiration and metabolism. Therefore, we propose that the insufficient ATP supply due to mitochondrial dysfunction caused by oxidative stress may contribute to embryonic development retardation.

Fetal growth and development can be improved with specific exogenous antioxidants targeting mitochondrial ROS [37]. Studies have highlighted the role of maternal Zn nutrition in the regulation of the expression of MTs and its role as a co-factor for antioxidant enzymes [9, 38]. As far as it is known to all, the protective effect of maternal Zn nutrition on mitochondrial oxidative stress in embryos is reported herein the first time. In the present study, it can be found that maternal dietary Zn addition increases Zn contents in egg yolk and improved the embryonic survivability, as reported by several studies [9, 39]. It is assumed that GSH and MT4 depletions by oxidative stress reflect intracellular oxidation. Effectively, maternal Zn nutrition not only could promote MT4 and GSH contents and CuZnSOD activity in the embryonic liver, which could mitigate BHP-induced MMP decease, ROS generation and mitochondrial lipid peroxidation via maintaining redox homeostasis. In vitro studies also confirmed that the increased ROS generation in hepatocytes subjected to BHP incubation is reversed by a Zn pretreatment. Since increasing the MT4 concentration by Zn incubation could be expected to prepare cells for a potential oxidative insult [8]. Notably, Zn treatment significantly increases and reverses the hepatic MT4 content and protein expression in liver tissues and hepatocytes under the BHP-induced condition, leading to the suppression of the mitochondrial oxidative damage. Other studies on the protective effect of Zn on oxidative stress induced by chlorpyrifos [40], and methomyl [41] have also reported similar data. With the increased mitochondrial membrane permeability, it is observed that the addition of Zn has a strong stimulating effect on mitochondrial function, causing an increase of ATP content in embryonic liver, possibility that Zn may stimulate the electron transport and oxidative phosphorylation systems in the mitochondria [42]. The MTs have been found in the intermembrane space of mitochondria where the concentration of ATP45 is high [43]. The interactions between ATP and MTs initiate the formation of a Zn-ATP complex as a specific cofactor of the key enzymes in energy metabolism [44]. Nevertheless, it can be inferred that maternal Zn nutrition plays a great role in enhancing the ability of MT synthesis and restore mitochondrial structure and function destroyed by oxidative stress during embryonic development.

The capacity of Zn to regulate antioxidant protective responses is in relevant in sustaining the cell redox homeostasis through various mechanisms [14], which have not yet been fully elucidated. Nrf2 was known to be a redox-sensitive transcriptional factor, which activates the expression of target genes to cope with cellular stresses by binding to antioxidant response elements, that are involved in detoxification and antioxidant defense to reduce oxidative stress [16]. In ovo BHP-induced oxidative stress decreased the mRNA and protein levels of Nrf2 and MT4 in embryonic liver, confirming that the Fe-induced mitochondrial oxidative stress involved directly inhibits the Nrf2 binding to antioxidant response elements. It was indicated that ROS accumulation contributed to inhibition of the Nrf2 expression and the promotion of its ubiquitin-mediated degradation in cells [45]. However, inconsistent with the results of in vivo, this paper observes a significant increase of abundance of the MT4 and Nrf2 in vitro, implying that the stressed response displays a model-dependent manner. Nevertheless, Zn deficiency inhibits the Nrf2 and MT4 expression both in the embryos and cells, aggravating the mitochondrial oxidative stress induced by BHP. It has been confirmed that Zn would act as an intracellular second messenger mediating the Nrf2 sensing of environmental stressors to activate MTs expressions in cells [16]. Consistent with this, maternal Zn addition alleviates BHP-induced the decrease of the Nrf2 and MT4 gene and protein expression levels in the embryonic liver. These data implied that Zn supplementation can protect the embryos from mitochondrial oxidative damage by regulating Nrf2 for the induction and expression of MT4, but the exact mechanism needs further study. This research further explores the underlying mode of Zn to improve mitochondrial biogenesis and energy metabolism. It has been shown that PGC-1α is critically involved in regulating mitochondrial respiration [46]. Notably, the present study reveals that Zn treatment could enhance mitochondrial biogenesis, which can be proved by reversing the alteration of mtDNA copy number, and upregulating the expression of PGC-1α and PPAR-α subjected to oxidative stress induced by BHP treatment in embryos and hepatocytes. In vivo studies have proven that Zn supplementation attenuates alcohol-mediated mitochondrial dysfunction in association with accelerated hepatic fatty acid oxidation by restoring PGC-1α signaling [14]. To sum up, the protective effects of Zn on BHP-evoked mitochondrial oxidative damage and dysfunction involve the activation of Nrf2/PGC-1α pathway.

## Conclusions

In conclusion, this study provides the latest information for the first time that maternal Zn nutrition protects mitochondrial oxidative stress in favor of embryonic development. The BHP-induced mitochondrial oxidative stress triggers the hepatic accumulation ROS and lipid peroxidation, aggravates mitochondrial damage and dysfunction, and finally increases the embryonic mortality. The Zn addition can effectively scavenge mitochondrial ROS and increase mitochondrial MMP and ATP content to mitigate the adverse effect via enhancing the antioxidant capacity and mitochondrial function. The findings provide about the mechanisms involved in the protective effect of Zn on mitochondrial oxidative stress contributing to the increase of MT4 synthesis and activation of Nrf2/PGC-1α signaling.

## Supplementary Information

**Additional file 1:** **Table S1.** Composition and nutrient levels of the semi-purified basal diet for laying broiler breeders during the Zn depletion period and experimental period (as-fed basis).

**Additional file 2:** **Table S2.** Nucleotide sequences of specific primers.

**Additional file 3:** **Table S3.** Summary of the antibodies used for Western Blot.

**Additional file 4:** **Table S4.** Effect of dietary Zn level on laying performance of broiler breeders.

## Data Availability

Data will be made available on request.

## Abbreviations

ATP: Adenosine triphosphate
ATP6: Adenosine triphosphate synthase 6
BCA: Bicinchoninic acid assay
BHP: *Tert*-Butyl hydroperoxide
BSA: Bovine serum albumin
CCK8: Cell counting Kit-8
CPT-1: Carnitine palmitoyl transterase-1
CPT-2: Carnitine palmitoyl transterase-2
CuZnSOD: Copper zinc superoxide dismutase
DCFH-DA: Dichlorodihydrofluorescein diacetate
EGTA: Ethylene glycol tetraacetic acid
FMN: Flavin mononucleotide
GC–MS: Gas chromatography-mass spectrometer
Gpx: Glutathione peroxidase
GSH: Glutathione
HEPES: N-2-hydroxyethylpiperazine-N-ethane-sulphonicacid
H&E: Hematoxylin and eosin
LC–MS: Liquid chromatograph mass spectrometer
MDA: Malondialdehyde
MMP: Mitochondrial membrane potential
MSD: Mitochondrial swelling degree
MT1: Metallothionein 1
MT4: Metallothionein 4
mtDNA: Mitochondrial DNA
NAD: Nicotinamide adenine dinucleotide
NADPH: Nicotinamide adenine dinucleotide phosphate
Nrf2: Nuclear factor erythroid-2 related factor 2
OXPHOS: Oxidative phosphorylation
PBS: Phosphate buffer saline
PGC-1α: Peroxisome proliferator-activated receptor γ coactivator-1α
PPAR-α: Peroxisome proliferators-activated receptor-α
PVDF: Polyvinylidene difluoride
ROS: Reactive oxygen species
RT-qPCR: Real time—quantitative PCR
SDS-Gel: Sodium dodecyl sulfte polyacrylamide gel electrophoresis
TCA: Tricarboxylic acid cycle
TFAM: Mitochondrial transcription factor A
VDAC: Mitochondrial voltage-dependent anion channel
Zn: Zinc
8-OHdG: 8-hydroxy-2-deoxyguanosine
