# Correction: Maternal zinc alleviates *tert*-butyl hydroperoxide-induced mitochondrial oxidative stress on embryonic development involving the activation of Nrf2/PGC-1α pathway

**DOI:** 10.1186/s40104-025-01179-9

**Published:** 2025-03-17

**Authors:** Liang Huang, Wei Gao, Xuri He, Tong Yuan, Huaqi Zhang, Xiufen Zhang, Wenxuan Zheng, Qilin Wu, Ju Liu, Wence Wang, Lin Yang, Yongwen Zhu

**Affiliations:** 1 State Key Laboratory of Livestock and Poultry Breeding, South China Agricultural University, Guangzhou, 510000 China; 2 Tongren Polytechnic College, Tongren, 554000 China; 3 Enping Long Industrial Co. Ltd, Enping, 529400 China

**Correction: J Animal Sci Biotechnol 14, 45 (2023)**

**https://doi.org/10.1186/s40104-023–00852-1**

Following publication of the original article [1], the authors reported that Fig. 3 were incorrect because there was naming error occurred during the archiving of electron microscopy micrographs of mitochondrial ultrastructure for maternal Zn treatment referring to Zn + pbs and Zn + BHP groups, resulting in the incorrect use of these images in Fig. 3D. The other elements of the Fig. 3D remain the same, and the interpretation of the results remains unchanged. This error does not affect the conclusions drawn in the paper.

Figure 3 is corrected from:

**Fig. 3:**
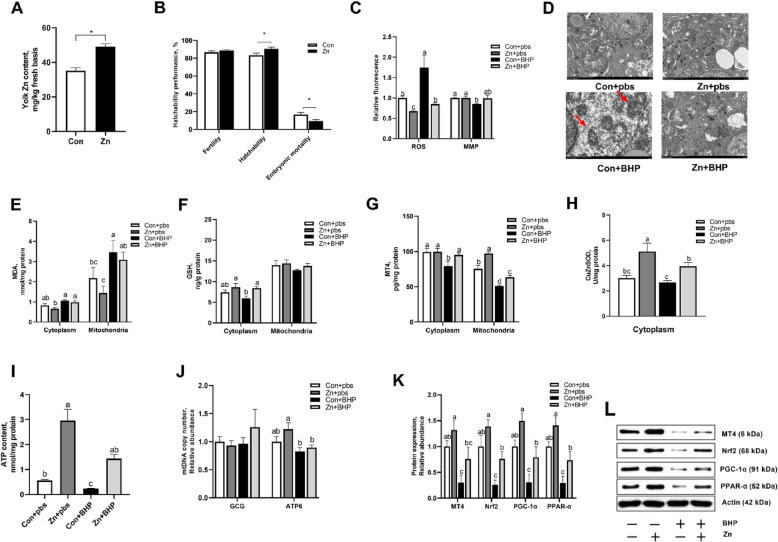
Maternal Zn addition attenuated in ovo injected BHP-induced mitochondrial dysfunction in embryo. The maternal Con and Zn groups diets were supplemented with either 0 or 220 mg Zn/kg diet for female broiler breeders. The embryos from Con and Zn groups were subjected to in ovo injection of either pbs or 600 μmol/L BHP on E14.** A** Effect maternal Zn addition on egg yolk Zn content. **B** Effect maternal Zn addition on hatchability performance. **C** Effect maternal Zn addition and in ovo injected BHP treatment on mitochondrial ROS and MMP. **D **Representative electron microscopy micrographs of mitochondrial ultrastructure. **E–G** Effect maternal Zn addition and in ovo injected BHP treatment on MDA, GSH, and MT4 contents in isolated cytoplasm and mitochondria. **H** Effect maternal Zn addition and in ovo injected BHP treatment on CuZnSOD activity in isolated cytoplasm. **I–J** Effect maternal Zn addition and in ovo injected BHP treatment on hepatic ATP content and mtDNA copy number. **K** and **L** Effect maternal Zn addition and in ovo injected BHP treatment on hepatic MT4, Nrf-2, PGC-1α, PPAR-α protein expressions. Graph bars in A and B were analyzed using unpaired two-tailed Student’s *t*-test (^∗^*P* < 0.05, *n* = 6), while graph bars in **C**, **E**, **G**, **H**,** I** and** J** marked with different letters on top represent statistically significant results (*P *< 0.05, *n *= 4–6) based on Tukey’s post hoc analysis, whereas bars labelled with the same letter correspond to results that show no statistically significant differences. Data were mean ± SEM

To: The original article [1] has been updated.

**Fig. 3:**
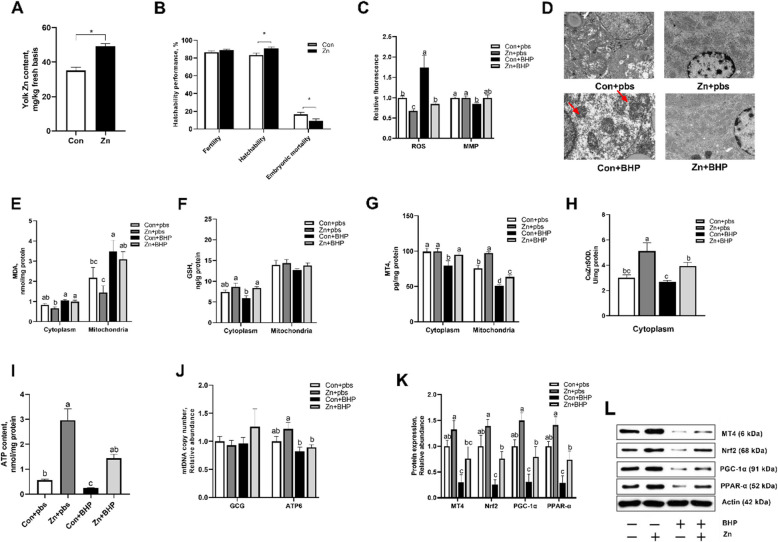
Maternal Zn addition attenuated in ovo injected BHP-induced mitochondrial dysfunction in embryo. The maternal Con and Zn groups diets were supplemented with either 0 or 220 mg Zn/kg diet for female broiler breeders. The embryos from Con and Zn groups were subjected to in ovo injection of either pbs or 600 μmol/L BHP on E14.** A** Effect maternal Zn addition on egg yolk Zn content. **B** Effect maternal Zn addition on hatchability performance. **C** Effect maternal Zn addition and in ovo injected BHP treatment on mitochondrial ROS and MMP. **D **Representative electron microscopy micrographs of mitochondrial ultrastructure. **E–G** Effect maternal Zn addition and in ovo injected BHP treatment on MDA, GSH, and MT4 contents in isolated cytoplasm and mitochondria. **H** Effect maternal Zn addition and in ovo injected BHP treatment on CuZnSOD activity in isolated cytoplasm. **I–J** Effect maternal Zn addition and in ovo injected BHP treatment on hepatic ATP content and mtDNA copy number. **K** and **L** Effect maternal Zn addition and in ovo injected BHP treatment on hepatic MT4, Nrf-2, PGC-1α, PPAR-α protein expressions. Graph bars in A and B were analyzed using unpaired two-tailed Student’s *t*-test (^∗^*P* < 0.05, *n *= 6), while graph bars in **C**, **E**, **G**, **H**,** I** and** J** marked with different letters on top represent statistically significant results (*P *< 0.05, *n *= 4–6) based on Tukey’s post hoc analysis, whereas bars labelled with the same letter correspond to results that show no statistically significant differences. Data were mean ± SEM
