# Supplementary material for: Maternal zinc alleviates tert-butyl hydroperoxide-induced mitochondrial oxidative stress on embryonic development involving the activation of Nrf2/PGC-1α pathway
Source: J Anim Sci Biotechnol. 2023 Apr 12;14:45. doi: 10.1186/s40104-023-00852-1 (PMC10091542; doi:10.1186/s40104-023-00852-1)
Supplement: Supplementary file 1 — Additional file 1: Table S1. Composition and nutrient levels of the semi-purified basal diet for laying broiler breeders during the Zn depletion period and experimental period (as-fed basis). [file 40104_2023_852_MOESM1_ESM.docx]

**Table S1** Composition and nutrient levels of the semi-purified basal diet for laying broiler breeders during the Zn depletion period and experimental period (as-fed basis)

| **Item** | **Percentage, %** |
| --- | --- |
| Ingredient |  |
| Corn | 51.50 |
| Corn starch | 19.30 |
| Corn gluten meal | 12.90 |
| Soybean meal | 5.00 |
| CaCO_3_ | 8.20 |
| CaHPO_4_ | 1.60 |
| NaCl | 0.30 |
| DL-Met (98%) | 0.40 |
| *L*-Lys·HCl | 0.35 |
| Micronutrients^a^ | 0.41 |
| Nutrient composition, % |  |
| ME^b^, MJ/kg | 11.99 |
| CP^c^ | 15.82 |
| Lys^b^ | 0.88 |
| Met^b^ | 0.47 |
| Met + Cys^b^ | 0.73 |
| Thr^b^ | 0.52 |
| Ca^c^ | 3.45 |
| Nonphytate P^c^ | 0.46 |
| Zn^c^, mg/kg | 26.34 |

^a^ Provided per kilogram of diet: vitamin A (retinyl acetate), 11,000 IU; cholecalciferol, 3,500 IU; vitamin E (α-tocopherol acetate), 50 IU; menadione, 4.40 mg; thiamin, 6.60 mg; riboflavin, 12.0 mg; pyridoxine, 4.50 mg; cyanocobalamin, 0.02 mg; pantothenate, 15.5 mg; niacin 50.0 mg; folic acid 2.0 mg; biotin, 0.22 mg; choline (choline chloride), 2,000 mg; Cu (CuSO_4_·5H_2_O), 10.0 mg; Fe (FeSO_4_·7H_2_O), 50.0 mg; Mn (MnSO_4_·H_2_O), 120 mg; I (KI), 1.20 mg; Se (Na_2_SeO_3_), 0.30 mg; Mo (NaMoO_4_·2H_2_O), 8.30 mg

^b^ Calculated values

^c^ Analysed values based on triplicate determinations
