# Supplementary material for: Maternal zinc alleviates tert-butyl hydroperoxide-induced mitochondrial oxidative stress on embryonic development involving the activation of Nrf2/PGC-1α pathway
Source: J Anim Sci Biotechnol. 2023 Apr 12;14:45. doi: 10.1186/s40104-023-00852-1 (PMC10091542; doi:10.1186/s40104-023-00852-1)
Supplement: Supplementary file 2 — Additional file 2: Table S2. Nucleotide sequences of specific primers. [file 40104_2023_852_MOESM2_ESM.docx]

**Table S2** Nucleotide sequences of specific primers

| **Target gene** | **Gene bank accession no.** | **Sequence (5’→3’)** | **Applications** |
| --- | --- | --- | --- |
| *CPT2* | NM_001031287.2 | F: GCTGGATAATGGCAATGAGGAAG  R: CAAGTTTCTGTACTGCTGTGGAA | RT-qPCR for gene mRNA quantification |
| *CPT1* | NM_001012898.1 | F: TGTAAAGAAGGCATAAAGG  R: CTGGTTGTAGAAAGGGAG |  |
| *Nrf-2* | [NM_205117.1](https://www.ncbi.nlm.nih.gov/nuccore/NM_205117.1) | F: CTGCCCAAAACTGCCGTA  R: CAAATCTTGCTCCAGTTCCA |  |
| *MT4* | NM_205275.1 | F: GGCGAAAGTCAACGAGAA  R: GCCCACGATAAGGAAACC |  |
| *SOD1* | [NM_205064.1](https://www.ncbi.nlm.nih.gov/nuccore/NM_205064.1) | F: TGTGCATGAATTTGGAGACAAC  R: TTGCAGTCACATTGCCGAG |  |
| *Gpx* | NM_001163245 | F: TTGTAAACATCAGGGGCAAA R: TGGGCCAAGATCTTTCTGTAA |  |
| *TFAM* | NM_204100.1 | F: AGCAGAACCCAGAACTGAAC  R: CAAGCACAGTCAATTCTCTC |  |
| *VDAC1* | NM_001033869.2 | F: TCAGCTGCCACATGATAGCAA  R: ACATCAAACCTTCTCGGCAAC |  |
| *PPAR-α* | NM_001001464.1 | F: TGTGGAGATCGTCCTGGTCT  R: CGTCAGGATG GTTGGTTTGC |  |
| *PGC-1α* | [NM_001006457.1](https://www.ncbi.nlm.nih.gov/nuccore/NM_001006457.1) | F: AAGCAATTGAAGAGCGTCGTG  R: TGTGCACTCCTCGATTTCACC |  |
| *β-actin* | NM_205518.1 | F: ACCTGAGCGAAGTACTCTGTCT  R: CATCGTACTCCTGCTTGCTGAT |  |
| *ATP6* | NC_040970.1 | F: ATTCTCAAGCCCCTGCCTAC  R: TCAGAGTTGGATGGTGGAGAGG | RT-PCR for mtDNA copy number |
| *GCG* | NC_006094.5 | F: GTGGAGGGCTGATAAAACACAAT R: TCCAACTCCTTGACCTCTATCC |  |

CPT-1, carnitine palmitoyl transterase-1; CPT-2, carnitine palmitoyl transterase-2; Gpx, glutathione peroxidase; MT1, metallothionein 1; MT4, metallothionein 4; Nrf2, nuclear factor erythroid-2 related factor 2; TFAM, mitochondrial transcription factor A; CuZnSOD, copper zinc superoxide dismutase 1; VDAC1, mitochondrial voltage-dependent anion channel 1 protein; PGC-1α, Peroxisome proliferator-activated receptor-γ coactivator 1-α; PPAR-α, peroxisome proliferators-activated receptor-α; ATP6, ATPase synthase; F, forward; R, reverse
