# Supplementary material for: Maternal zinc alleviates tert-butyl hydroperoxide-induced mitochondrial oxidative stress on embryonic development involving the activation of Nrf2/PGC-1α pathway
Source: J Anim Sci Biotechnol. 2023 Apr 12;14:45. doi: 10.1186/s40104-023-00852-1 (PMC10091542; doi:10.1186/s40104-023-00852-1)
Supplement: Supplementary file 3 — Additional file 3: Table S3. Summary of the antibodies used for Western Blot. [file 40104_2023_852_MOESM3_ESM.docx]

**Table S3** Summary of the antibodies used for Western Blot

| **Antibodies** | **Molecular weight, kDa** | **Host species** | **Source** | **Catalogue no.** | **Dilution** |
| --- | --- | --- | --- | --- | --- |
| MT4 | 6 | Rabbit | Bioss | bs-1328R | 1:1000 |
| Nrf-2 | 68 | Rabbit | Proteintech | 16396-1-AP | 1:1000 |
| PGC-1α | 91 | Mouse | Proteintech | 66369-1-Ig | 1:5000 |
| PPAR-α | 52 | Rabbit | Proteintech | 15540-1-AP | 1:750 |
| β-actin | 42 | Mouse | Proteintech | 66009-1-Ig | 1:5000 |

MT4, metallothionein 4; Nrf2, nuclear factor erythroid-2 related factor 2; PGC-1α, Peroxisome proliferator-activated receptor-γ coactivator 1-α; PPAR-α, peroxisome proliferators-activated receptor-α
