# Supplementary material for: Maternal zinc alleviates tert-butyl hydroperoxide-induced mitochondrial oxidative stress on embryonic development involving the activation of Nrf2/PGC-1α pathway
Source: J Anim Sci Biotechnol. 2023 Apr 12;14:45. doi: 10.1186/s40104-023-00852-1 (PMC10091542; doi:10.1186/s40104-023-00852-1)
Supplement: Supplementary file 4 — Additional file 4: Table S4. Effect of dietary Zn level on laying performance of broiler breeders. [file 40104_2023_852_MOESM4_ESM.docx]

**Table S4** Effect of dietary Zn level on laying performance of broiler breeders

| **Dietary Zn treatment** | **Egg weight, g** | **Laying rate, %** | **Egg mass, g/bird/d** | **Feed intake, g/bird/d** | **Feed:egg,**  **g:g** |
| --- | --- | --- | --- | --- | --- |
| Con | 40.59 | 54.37 | 27.66 | 79.70 | 2.99 |
| Zn | 41.06 | 53.87 | 27.71 | 80.11 | 2.89 |
| SEM | 0.27 | 1.40 | 0.80 | 0.62 | 0.06 |
| *P*值 | 0.40 | 0.71 | 0.96 | 0.41 | 0.15 |

Con, maternal Zn-deficient group (0 mg Zn/kg diet); Zn, maternal Zn-adequate group (220 mg Zn/kg diet)
